# Saccades and Vergence Performance in a Population of Children with Vertigo and Clinically Assessed Abnormal Vergence Capabilities

**DOI:** 10.1371/journal.pone.0023125

**Published:** 2011-08-09

**Authors:** Maria Pia Bucci, Zoï Kapoula, Emmanuel Bui-Quoc, Aurelie Bouet, Sylvette Wiener-Vacher

**Affiliations:** 1 Laboratoire de Psychologie et Neuropsychologie Cognitives, FRE 3292 CNRS IUPDP Université Paris Descartes, Boulogne Billancourt, France; 2 Centre d'études de la sensori-motricité (CESM), UMR 8194 CNRS, Paris, France; 3 Service d'OPH, Hôpital Robert Debré, Paris, France; 4 Service d'ORL, Hôpital Robert Debré, Paris, France; Tokyo Metropolitan Institute of Medical Science, Japan

## Abstract

**Purpose:**

Early studies reported some abnormalities in saccade and vergence eye movements in children with vertigo and vergence deficiencies. The purpose of this study was to further examine saccade and vergence performance in a population of 44 children (mean age: 12.3±1.6 years) with vertigo symptoms and with different levels of vergence abnormalities, as assessed by static orthoptic examination (near point of convergence, prism bar and cover-uncover test).

**Methods:**

Three groups were identified on the basis of the orthoptic tests: group 1 (n = 13) with vergence spasms and mildly perturbed orthoptic scores, group 2 (n = 14) with moderately perturbed orthoptic scores, and group 3 (n = 17) with severely perturbed orthoptic scores. Data were compared to those recorded from 28 healthy children of similar ages. Latency, accuracy and peak velocity of saccades and vergence movements were measured in two different conditions: gap (fixation offset 200 ms prior to target onset) and simultaneous paradigms. Binocular horizontal movements were recorded by a photoelectric device.

**Results:**

Group 2 of children with vergence abnormalities showed significantly longer latency than normal children in several types of eye movements recorded. For all three groups of children with vergence abnormalities, the gain was poor, particularly for vergence movement. The peak velocity values did not differ between the different groups of children examined.

**Interpretation:**

Eye movement measures together with static orthoptic evaluation allowed us to better identify children with vergence abnormalities based on their slow initiation of eye movements. Overall, these findings support the hypothesis of a central deficit in the programming and triggering of saccades and vergence in these children.

## Introduction

Vertigo is a common symptom of vestibular dysfunction or visual disorders [1]. Anoh-Tanon et al. [2] first reported that a large number of children consulting the ENT (Ear Nose Throat) department for vertigo and headaches showed normal vestibular function but had vergence abnormalities. These authors suggested that vergence deficits could make gaze stabilization during body movements difficult, and thus cause double or blurry vision, which can lead to vertigo symptoms. Despite the convincing clinical evidence of the link between vertigo symptoms and vergence abnormalities, few studies have examined such abnormalities with objective measures. Several studies by our group had the aim of substantiating this clinical observation with vergence and saccade eye movement recordings. Bucci et al. [3] studied a small group of 12 children suffering from vertigo and headaches, who had normal vestibular function but showed signs of vergence abnormalities, as assessed by orthoptic tests, and reported abnormally slow latency for saccades and vergence eye movements. In the same study, we examined the effect of orthoptic vergence training after 12 sessions of vergence exercises; latency was found to have improved but convergence remained above the normal level. In a subsequent study, Bucci et al. [4] reported abnormalities affecting the accuracy and mean speed of vergence movements; we attributed this deficit to downstream brain stem function. Again, orthoptic vergence training improved the accuracy of vergence movements but performance remained under the normal level. In another study by Bucci et al. [5], the binocular coordination of saccades in 15 children with vertigo was examined at far and near distances. The binocular coordination of saccades at far distance was normal; at near distance, however, it was abnormal. Some of these children were examined again after 12 sessions of orthoptic vergence training, and the disconjugacy of their saccades at near distance had improved considerably, dropping to normal values. All these findings support the hypothesis that these deficiencies could be due to dysfunctions of the cortical and/or subcortical structures controlling eye movements. Orthoptic training, perhaps acting via attentional and motor mechanisms, may improve vergence performance, and thereby allow for improved eye movements.

In the present study, we further examined saccade and vergence movements in a similar population. We collected data from 44 children who were referred to the ENT or Ophthalmology department for vertigo and headaches and who had normal vestibular function but orthoptic vergence abnormalities. Based on these clinical and subjective findings, the children were classified in three different groups showing low, intermediate and high levels of vergence abnormalities (according to the classification done in our previous studies [6],[7]). Data were compared with those obtained from a group of 28 normal children of comparable ages. Our intent was to examine whether eye movement recordings could be an objective tool to better discriminate these different groups of children; briefly, we wondered whether we would find signs of different oculomotor behavior for the children with clinically assessed low, intermediate and high levels of vergence abnormalities. In other words, we aimed to further investigate the link between orthoptic results and eye movement performance in this population of children.

Furthermore, in order to explore whether children with vertigo could benefit from the gap paradigm, the gap and simultaneous paradigms were used to elicit saccades and vergence movements. To perform an eye movement, several cortical and subcortical areas are activated: the visual information from the retina is sent to the visual cortex, parietal cortex, frontal lobe, and superior colliculus; from there, via the brain stem, the motor command is sent to the extra-ocular muscles [8]. It is well known that during the preparation of an eye movement several processes take place, such as shifting of visual attention to the new target, disengagement of oculomotor fixation, and computation of the parameters of the movement [9],[10]. Saslow [11] was the first to show that the latency of eye movements is influenced by the temporal relationship between the offset of the fixation point and the onset of the target. Indeed, the latency of eye movements is shortened in the gap paradigm, in which a delay (200 ms) is introduced between those two points. The “gap effect” is described in the literature as the decrease in eye movement latency that occurs in the gap paradigm in comparison to the overlap paradigm, in which the fixation point remains present when the target appears [9]–[12].

The reduction in eye movement latency in the gap paradigm is most likely due to facilitation of the disengagement of fixation and attention [13],[14]. This idea is integrated in models such as that of Findlay and Walker [10], who suggested that during the stimulus offset there is a decrease in fixation activity, promoting rapid movement initiation. The objective here was to evaluate whether the gap paradigm could also lead to better eye movement performance, in terms not only of latency but also of precision and peak velocity of eye movements, in children with vergence abnormalities.

To summarize, we made the hypothesis that in children with vergence abnormalities the gap effect is working as in normal children, leading to facilitation of the triggering and the execution of eye movements. In this case our children population could benefit from the gap paradigm and this maybe has important implication for developing new training procedures based on visuo-attentional processing.

Finally, in this study, we wished to address the question of whether a distinct group of children with vergence abnormalities has a specific impairment affecting eye movements, and potentially to suggest new vergence training techniques specific to the vergence abnormalities clinically observed in these children.

## Methods

### Subjects

Forty-four children from 10 to 15 years old participated in the study. The mean age was 12.3±1.6 years. The children had been referred to the ENT and ophthalmology departments of the children's hospital for vertigo and headaches; they reported episodes when the environment seemed to be moving around them or when they felt dizzy or unbalanced. These symptoms occurred several times a day, were frequently related to fatigue (at school or at the end of the day, and often after long exposure to computer or television screens), and were usually brief, lasting less than 1 minute. None of the children had neurological problems and they were not taking any medication. All of the children underwent complete vestibular and orthoptic examinations, as described in earlier articles [6],[7]. Data from children with vertigo were compared with data from a group of 28 normal children of comparable ages (mean age 12.3±1.3 years) without any vertigo symptoms.

### Ethics statement

The investigation adhered to the principles of the Declaration of Helsinki and was approved by the local human experimentation committee (CPP IIe France V, Saint Antoine Hospital). Written consent was obtained from the children's parents after an explanation of the experimental procedure.

### Vestibular examination

Vestibular testing included a complete battery of tests for evaluation of canal and otolith function: caloric bithermal tests, earth vertical axis rotation, off vertical axis rotation with a computer-controlled rotating chair in the dark and VEMP (vestibular evoked myogenic potential [15],[16]). The evaluation of inner ear function was complemented by conventional hearing tests (tonal and speech audiometric techniques). The results of all these tests were normal for all the children participating in this study. Consequently, the vertigo symptoms reported by these children were not due to vestibular disorders.

### Ophthalmologic examination

All children underwent an ophthalmologic examination to evaluate their visual function. Visual acuity was evaluated for each eye separately at long (5 m) and short (30 cm) distances with the Monoyer chart [17] and the Parinaud scale, respectively.

The stereoacuity threshold based on disparity detection was tested with the TNO random dot test for stereoscopic depth discrimination (Netherlands Organisation, Richmond Products, Boca Raton, FL). The near point of convergence (NPC) was also examined by placing a small pen-light at 30 to 40 cm in the midplane in front of the child and moving it slowly towards the eyes until one eye lost fixation. Heterophoria (i.e., the latent deviation of one eye when the other is covered) was measured at both distances (far and near) by using the cover-uncover test and the Maddox test. Finally, convergence and divergence fusional amplitude were measured at both long (4 m) and short (30 cm) distances by using a base-in and a base-out prism bar. Divergence amplitude was measured twice, before and after the convergence measure, in order to evaluate accommodative spasm [18]. All these tests allowed us to assign the children with vertigo to three different groups (containing 13, 14 and 17 children, respectively), depending on their degree of vergence abnormalities, as described in earlier articles [6],[7].

Then refraction was measured under cycloplegia performed with instillation of cyclopentolate ¾ h after eye drops (cyclopentolate is a drug that temporarily paralyzes accommodation).

### Oculomotor paradigms

The procedure was similar to that used in our previous studies [19],[20]. The children faced a horizontal table in which eight red light-emitting diodes (LEDs) were embedded. The LEDs were arranged on two isovergence curves. Five LEDs were placed 150 cm from the children's eyes, one at the center, two at ±10° (used for the calibration task only) and two at ±20°. The required mean angle of vergence for fixating these diodes was 2.3°. Three other LEDs were placed at a distance of 20 cm, one at the center and two at ±20°; the mean angle of vergence was 17°. The fixation point was either the central LED at a distance of 20 cm or the central LED at a distance of 150 cm. Two types of movements were elicited: pure saccades to the left or to the right were elicited either at the close distance of 20 cm or at the far distance of 150 cm; pure vergence, either convergence or divergence, was elicited between the two LEDs placed on the median plane at 20 cm and at 150 cm. The required saccade amplitude was always 20°; the required vergence movement was always 15°.

**Figure 1:**
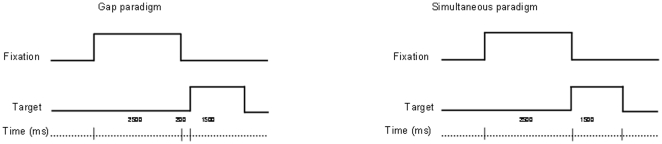
Schematic diagram of the temporal arrangement used in the two different paradigms (gap and simultaneous).

Two temporal paradigms were used (see Figure 1): the gap paradigm (to elicit short-latency eye movements) and the simultaneous paradigm (to favor more voluntary eye movements). For each trial, the central LED (at a distance of either 20 cm or 150 cm) was switched on for a period of 2.5 s. Then it was switched off, and a target LED appeared 200 ms later (gap period). The target LED stayed on for 1.5 s. A delay of 0.5 s was introduced before the next trial. In the simultaneous paradigm, after a 2.5-s fixation period, the central LED was switched off and the target LED was simultaneously switched on for 1.5 s. A delay of 0.5 s was introduced before the next trial. The instruction given to the child was to look at the target LED as accurately and as rapidly as possible.

### Design

The child was in a dark room, seated in a chair with the head stabilized by a forehead and chin support. He/she faced the horizontal surface where the LEDs were positioned at eye level; viewing was binocular. Each child performed four blocks of 24 trials each, two with the gap and two with the simultaneous paradigm; blocks of gap and simultaneous paradigms were randomly ordered; each block was followed by a few minutes of rest.

In each block, the two types of trials (saccades and vergence movements) were interspersed randomly. Each block contained six saccades at the far viewing distance (three rightwards and three leftwards), six saccades at the close viewing distance (three rightwards and three leftwards), six convergence trials (along the median plane), and six divergence trials. Calibration was done before and after each block.

### Calibration task

Children performed a sequence of saccades to the target LED, moving from 0° to ±10° or 0° to ±20° on the far isovergence circle. During each of these trials, the target LED remained lit at each location for 2 s. Children were instructed to fixate the target LED as accurately as possible; the duration of the target LED presentation was sufficiently long to allow accurate and stable fixation. The calibration factors were extracted from these recordings.

### Eye movement recordings

Data collection was controlled with REX software (provided online at http://www.tchain.com by Timothy C. Hain, Northwestern University Medical School, Chicago, IL). Horizontal eye movements from both eyes were recorded simultaneously with a photoelectric device, Oculometer Dr. Bouis (Karlsruhe, Germany). This system has a resolution of 2 minutes of arc and a linear range of 20° [21]. Eye-position signals were lowpass-filtered with a cutoff frequency of 200 Hz and digitized with a 12-bit analogue-to-digital converter; each channel was sampled at 500 Hz.

### Data analysis

Our calibration and analysis methods were similar to those used in prior studies [3],[4],[20]. Briefly, calibration factors for each eye were extracted from the eye positions during the calibration procedure; a polynomial function was used to fit the calibration data. From the two individual eye position signals, we calculated the conjugate saccadic signal [(left eye+right eye)/2] and the disconjugate vergence signal (left eye – right eye). Markers were placed automatically at different points of the eye position signals. The onset of the conjugate saccadic component was defined as the time when the eye velocity reached 5% of the saccadic peak velocity. The offset of this signal was defined as the time when the eye velocity dropped below 10 deg/s. The onset and the offset of the vergence signals were defined as the time point when the eye velocity exceeded or dropped below 5 deg/s. These criteria are standard and similar to those used by other authors [22].

Statistical analysis corresponded to a mixed ANOVA with the different groups of children (normal children and three subgroups of children with vertigo and different levels of vergence abnormalities) as between-subject factor and the clinical findings reported by the orthoptic tests as within-subject factor. A similar ANOVA was also run on the individual means for the latency, occurrence of express movements, gain and peak velocity of each type of eye movement recorded in the two different conditions (gap and simultaneous) as within-subject factors. Post hoc comparisons were done with the least significant differences (LSD) method; the effect of a factor is significant when the *P*-value is below 0.05.

## Results

### Symptoms

The symptoms (type of vertigo or dizziness, nausea) were evenly distributed among the three groups of children distinguished on the basis of orthoptic clinical static examination. In the three subgroups of children with vertigo and mild, intermediate or severe levels of vergence abnormalities, headaches were reported by 46%, 78% and 58% of the children, respectively.

### Visual evaluation

The corrected visual acuity was normal at both long and short distances (≥20/25). All children had normal binocular vision (60 sec of arc or better), as evaluated with the TNO random dot test for stereoscopic depth discrimination. For normal children without vertigo symptoms (see Table 1), the NPC, phoria and vergence fusional amplitude values at both distances tested were normal. In contrast, several children with vertigo had abnormal values for NPC, phoria and vergence fusional amplitude (see Table 2). Children with vertigo were assigned to three diagnostic groups showing different signs of vergence abnormalities, based on the classification done previously in a similar population of children [6],[7].

- **Group 1: low level of vergence insufficiency** with the presence of accommodative spasms from V1 to V13; 46% of children complained of headaches;
- **Group 2: intermediate level of vergence insufficiency** (V14–V27) due to abnormally distant NPC (≥7 cm) in the case of five children, poor fusional convergence at long distance and abnormal phoria values at short distance (for children V17, V18, V20, V21, V23 and V24). For the majority of this group of children, poor convergence at a short distance was also observed, and 78% of them complained of headaches;
- **Group 3: high level of vergence insufficiency** due to abnormally distant NPC (≥7 cm) in the case of six children, poor fusional divergence and convergence at both distances. For several of these children, exophoria at both distances was also reported (V28–V44); 58% of them complained of headaches.

**Table 1:** Characteristics of children without any vertigo symptoms.

Children(years)	NPC(cm)	Heterophoria(pD)		Divergence(pD)		Convergence(pD)	
		Far	Near	Far	Near	Far	Near
C1 (10)	5	0	0	6	12	25	55
C2 (11)	3	−2	−3	8	14	14	30
C3 (11)	3	0	−2	6	16	16	30
C4 (11)	5	−2	0	8	12	25	40
C5 (11)	4	0	−2	6	10	20	30
C6 (11)	5	1	0	8	14	16	30
C7 (11)	5	1	−2	6	12	40	40
C8 (11)	5	0	0	8	14	30	40
C9 (11)	4	1	−2	6	12	35	60
C10 (12)	3	0	2	8	18	40	40
C11 (12)	4	−2	−3	6	16	18	30
C12 (12)	3	0	−2	8	12	18	30
C13 (12)	5	−2	0	6	10	16	35
C14 (12)	5	1	−2	12	16	20	40
C15 (12)	4	0	−2	10	18	18	30
C16 (12)	3	2	0	12	18	18	30
C17 (12)	5	0	0	8	16	25	40
C18 (12)	5	1	−2	10	16	16	30
C19 (13)	3	2	0	8	16	18	30
C20 (13)	4	0	−2	6	14	18	35
C21 (13)	5	−2	−3	6	14	18	30
C22 (13)	5	0	1	6	12	45	60
C23 (14)	4	1	0	8	18	20	35
C24 (14)	5	−2	−2	6	12	18	35
C25 (14)	4	−2	−2	8	14	25	35
C26 (14)	4	1	−2	6	16	35	60
C27 (15)	4	−2	−2	8	14	35	55
C28 (15)	4	0	−4	8	14	25	50

Near point of convergence (NPC) is measured in centimeters; heterophoria and vergence fusion are measured with the orthoptic technique, and values are given in prism diopters. Heterophoria and vergence amplitude were measured at far (4 m) and near (30 cm) distances. Heterophoria is negative in the case of exotropia and positive in the case of esophoria.

**Table 2:** Characteristics of children with vertigo and vergence abnormalities.

Children(years)	NPC(cm)	Heterophoria(pD)		Divergence(pD)		Convergence(pD)		Vertigo	Headache
		Far	Near	Far	Near	Far	Near		
V1 (10)	4	−1	−4	2	8	12	40	rotatory	x
V2 (11)	5	−2	−4	2	10	6	40	rotatory	
V3 (11)	4	2	14	4	6	20	30	unsteadiness	x
V4 (11)	5	4	2	8	12	25	35	unsteadiness	
V5 (11)	5	0	2	2	8	20	40	unsteadiness	x
V6 (11)	5	−2	−6	2	6	12	40	rotatory	x
V7 (11)	5	−1	4	8	8	16	30	rotatory	x
V8 (12)	4	0	−4	6	10	20	30	rotatory	
V9 (12)	5	0	2	4	8	25	40	rotatory	
V10 (12)	5	0	0	4	6	12	40	unsteadiness	
V11 (13)	5	0	0	6	12	25	35	rotatory	
V12 (14)	5	0	0	8	15	20	30	rotatory	x
V13 (15)	5	0	2	1	6	20	40	rotatory	
V14 (10)	4	0	0	2	10	15	20	unsteadiness	x
V15 (10)	7	−2	−4	2	12	4	25	rotatory	x
V16 (11)	5	−2	−4	2	4	14	30	unsteadiness	x
V17 (11)	5	0	6	4	8	10	35	unsteadiness	x
V18 (11)	3	2	2	8	8	15	20	rotatory	x
V19 (11)	5	0	−2	2	6	14	25	unsteadiness	x
V20 (12)	15	0	−14	4	18	16	25	rotatory	
V21 (12)	5	2	2	4	10	16	20	rotatory	
V22 (13)	7	0	−4	2	16	18	30	unsteadiness	x
V23 (13)	5	0	6	6	14	15	20	unsteadiness	x
V24 (14)	3	0	3	6	12	15	25	unsteadiness	x
V25 (14)	7	0	0	8	12	12	30	rotatory	
V26 (14)	7	0	−4	2	8	20	25	rotatory	x
V27 (15)	3	0	0	1	6	16	35	rotatory	x
V28 (10)	7	−2	−4	2	6	2	12	unsteadiness	x
V29 (10)	7	−2	−2	4	10	10	16	rotatory	x
V30 (10)	5	0	−2	4	8	10	12	rotatory	
V31 (11)	10	0	0	1	10	12	16	rotatory	
V32 (11)	5	0	2	4	14	14	18	rotatory	x
V33 (11)	20	0	0	2	6	8	14	unsteadiness	
V34 (12)	5	−2	−6	2	8	2	12	rotatory	
V35 (12)	5	−2	2	4	8	6	16	unsteadiness	x
V36 (13)	5	1	2	2	2	2	12	rotatory	x
V37 (13)	7	−2	−4	2	8	2	14	unsteadiness	x
V38 (13)	7	0	2	4	12	12	20	rotatory	
V39 (14)	5	0	0	4	4	14	16	unsteadiness	x
V40 (14)	6	−2	−4	2	8	4	20	rotatory	x
V41 (14)	5	−4	−4	4	10	8	18	rotatory	
V42 (15)	6	0	−8	4	10	2	14	rotatory	x
V43 (15)	7	0	−8	2	8	6	10	unsteadiness	x
V44 (15)	5	−2	−4	4	8	6	25	unsteadiness	

Other notes are as in Table 1. The different sensations perceived by the children with vertigo are reported (rotatory translation, rolling sensations and unsteadiness), as are headaches.

In order to improve the range of vergence fusional amplitude, orthoptic training was prescribed for the majority of these children (36/44); for the other eight children (V1, V5, V9, V10, V15, V21, V23 and V39), a hypermetropic correction of 0.50 diopters for both eyes was first prescribed to see whether this correction of visual acuity would be sufficient to improve the child's comfort. Recall that the purpose of plus lenses is to decrease the demand on the accommodation system and/or to reduce the amount of esodeviation by manipulating the crosslink AC/A ratio [23]. Such a small correction was prescribed only for children who showed improvement in their visual acuity at far distances while wearing plus lenses. After training and/or after wearing plus lenses, none of the children felt vertigo or headaches.

The ANOVA shows a significant group effect for several orthoptic examinations: the NPC (F_(3,68)_ = 3.73, *P*<0.01), the divergence values at both distances (F_(3,68)_ = 24.63, *P*<0.0001 and F_(3,68)_ = 19.52, *P*<0.0001, for long and short distances respectively), and the convergence values at long and short distances (F_(3,68)_ = 23.69, *P*<0.0001 and F_(3,68)_ = 39.88, *P*<0.0001, respectively). Post hoc comparisons showed significantly larger values for divergence and convergence fusional amplitude at both distances in normal children compared to all three groups of children with vertigo; furthermore, the group 3 children (with high vergence abnormalities) showed significantly lower values for divergence and convergence fusional amplitude than the other two groups of children.

To sum up, clinical ophthalmologic evaluation showed poor vergence fusional amplitude for all three groups of children compared to normal children; group 3 also showed reduced vergence capabilities in comparison to the other groups of children.

Next we will present the latency, gain and peak velocity results for each subgroup of children with vertigo and for the control children.

### Latency

**Figure 2:**
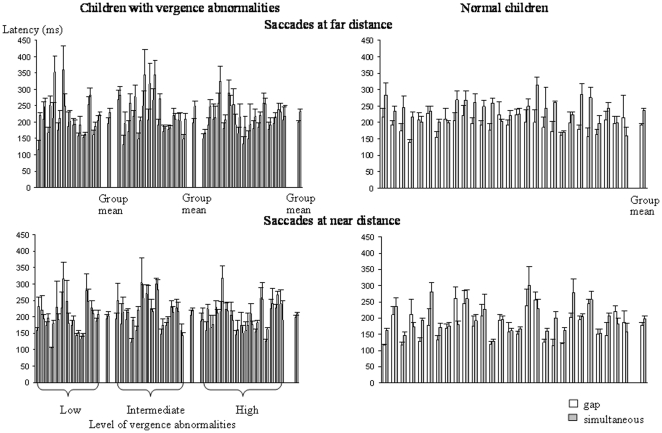
Mean latency of saccades at far and near distances in the gap (gray bars) and the simultaneous condition (white bars) for the three different groups of children with vertigo and different levels of vergence abnormalities and for control children. Group means are based on 13, 14 and 17 children for the children with vertigo and on 28 children for the control group. Vertical lines indicate the standard error.

**Figure 3:**
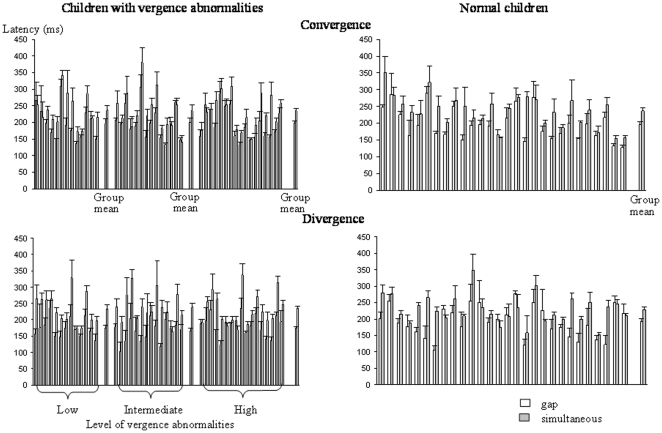
Mean latency of convergence and divergence in the gap (gray bars) and the simultaneous condition (white bars) for the three different groups of children with vertigo and different levels of vergence abnormalities and for control children. Other notes are as in Figure 2.

Values for latency of saccades at far and near distances are shown in Figure 2, and those for convergence and divergence in Figure 3. Data are shown for both the gap and simultaneous conditions, for the three groups of children with vertigo and different levels of vergence abnormalities and for the control children. Latency is highly variable between the different groups of children.

The ANOVA failed to show any significant effect of group of children (F_(3,68)_ = 0.19, *P* = 0.27), whereas there was a significant effect of condition (F_(1,68)_ = 139.02, *P*<0.001): latencies were longer in the simultaneous than in the gap condition. Furthermore, there was also a significant effect of the type of eye movement (F_(3,204)_ = 3.94, *P*<0.009). Importantly, the ANOVA showed a significant interaction between group and type of eye movement (F_(9,204)_ = 2.75, *P*<0.04). Post hoc comparisons showed significantly longer latency compared to control children, particularly in group 2 (children with an intermediate level of vergence abnormalities). Indeed, latency values for saccades at a near distance (in both gap and simultaneous conditions) and for divergence in the simultaneous condition were significantly longer than those recorded in control children.

The occurrence of express eye movements (movements with a latency value between 80 and 120 ms) was also calculated for the different groups of children. The ANOVA failed to show any significant effect of group (F_(3,68)_ = 0.04, *P* = 0.84), but showed a significant effect of paradigm (F_(1,68)_ = 17.21, *P*<0.001) and of type of movement (F_(3,204)_ = 23.05, *P*<0.001).

### Accuracy

**Figure 4:**
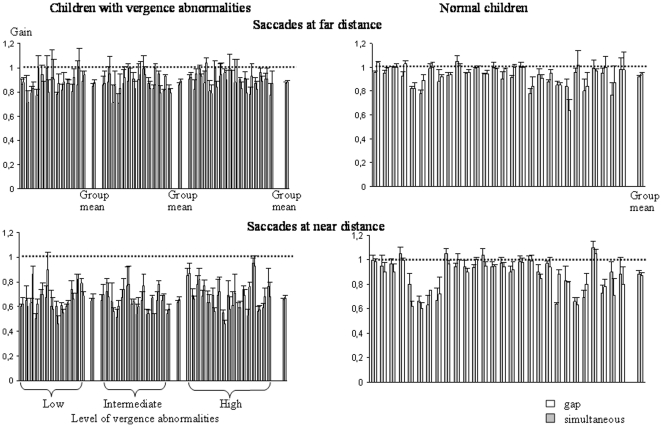
Mean gain (amplitude of the movement/target-LED excursion) of saccades at far and near distances in the gap (gray bars) and the simultaneous condition (white bars) for the three different groups of children with vertigo and different levels of vergence abnormalities and for control children. Other notes are as in Figure 2.

**Figure 5:**
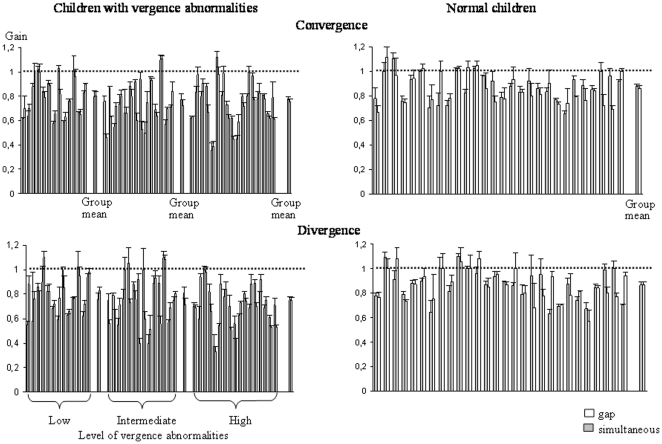
Mean gain of convergence and divergence in the gap (gray bars) and the simultaneous condition (white bars) for the three different groups of children with vertigo and different levels of vergence abnormalities and for control children. Other notes are as in Figure 2.

The gain (amplitude of the movement/target-LED excursion) of saccades at far and near distances is shown in Figure 4 and that of convergence and divergence in Figure 5. For all types of movements tested, the gain was poor for all groups of children with vergence abnormalities in comparison to control children; the gain for vergence movements was very low.

The ANOVA showed a significant effect of group (F_(3,68)_ = 15.00, *P*<0.001) and a significant effect of type of eye movement performed (F_(3,204)_ = 33.01, *P*<0.001). Furthermore, there was also a significant interaction between the groups of children examined and the types of eye movements (F_(9,204)_ = 4.03, *P*<0.001). Post hoc comparisons showed that the gain reported for convergence and divergence movements for the three groups of children with vertigo and vergence abnormalities was significantly lower than the other gains.

### Peak velocity

**Figure 6:**
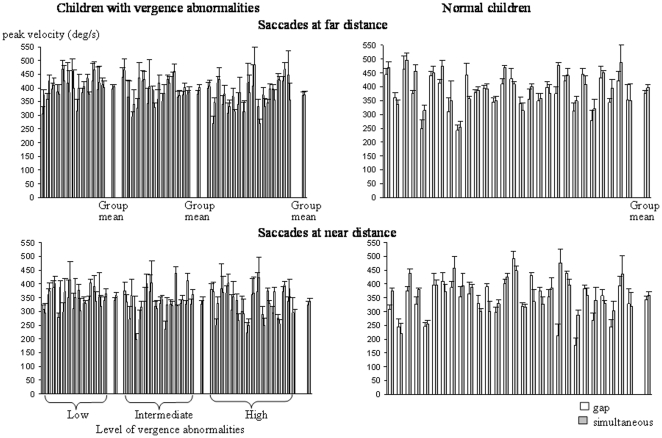
Mean peak velocity of saccades at far and near distance of saccades at far and near distance in the gap (gray bars) and the simultaneous condition (white bars) for the three different groups of children with vertigo and different levels of vergence abnormalities and for control children. Other notes are as in Figure 2.

**Figure 7:**
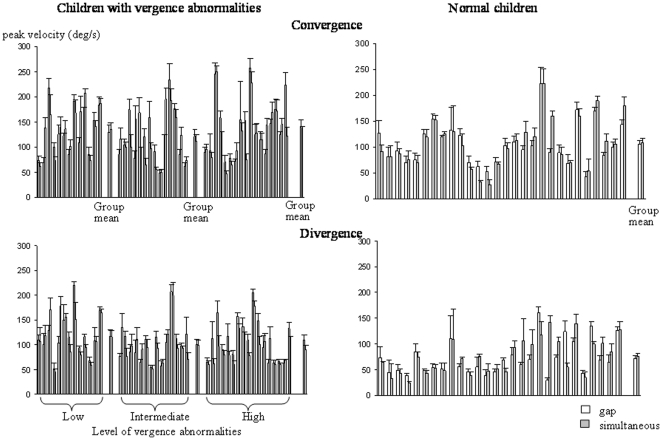
Mean peak velocity of convergence and divergence in the gap (gray bars) and the simultaneous condition (white bars) for the three different groups of children with vertigo and different levels of vergence abnormalities and for control children. Other notes are as in Figure 2.

The mean peak velocity of the different types of eye movements recorded is shown in Figures 6 and 7. The peak velocity was similar in the different groups of children examined and also in both conditions tested. The ANOVA failed to show a significant effect of group (F_(3,68)_ = 1.32, *P* = 0.2) or of condition (F_(1,68)_ = 2.13, *P* = 0.87); in contrast, we found a significant effect of the type of eye movement performed (F_(3,204)_ = 1070, *P*<0.001).

## Discussion

The main findings reported in the present study are as follows: (i) The gap effect, that is, shortening of mean latency and increase in the rate of express movements, was observed for both populations of children examined. (ii) For saccades at near distance and divergence eye movements, group 2 of children with vergence abnormalities showed significantly longer latency than normal children. (iii) All three groups of children with vergence abnormalities showed significantly poorer gain than controls particularly for vergence movements and regardless of the condition tested. (iv) Peak velocity values did not differ between the different groups of children examined. These findings will be discussed individually below.

### Gap effect

The first important finding of the present study is that the gap paradigm, for both groups of children (with and without vergence abnormalities), reduced significantly the mean latencies of saccades at far and at close distance, of convergence as well as divergence movements. This result is news because several studies examining the gap effect compared the gap paradigm *versus* overlap paradigm [24], [25], [26]. Here we compared gap paradigm versus simultaneous paradigm, however we found a relevant shortening of latencies for all types of eye movements tested. The mechanisms underlying the saccade latency reduction in the gap paradigm are still controversial. Indeed, as mentioned in the Introduction, for some authors [27], [28], the fixation offset occurring before the target onset may provide a general warning signal that could facilitate the detection of the target and the preparation of the movement through it. Other studies [13], [24] have emphasized the role of fixation offset which may provide a disengagement of both fixation and attention. We believes that the shortening of the latencies can be attributed to disengaging the fixation; according to Findlay and Walker [10] if such attentional disengagement occurs before the stimulus onset the fixation activity decrease, therefore accelerating movement initiation.

Importantly, such mechanism exists also for all groups of children with vergence abnormalities tested. According to this finding we could assume that fixation mechanisms are working correctly in such groups of children.

Furthermore, the present report shows that the gap condition is not a useful tool for discriminating among populations of children with vergence abnormalities, given that its effect (decreasing mean latency values and increasing the occurrence of express movements) was similar in all the children tested.

### Latency

It is well known that latency value depends on the subject's attention and motivation, particularly in the case of children [29]. Furthermore, latency of eye movements in children, which several authors have studied [24]–[26]–[30]–[31] has been shown to be longer in young children and to decrease with age. This has been attributed to the underdeveloped cortical structures involved in eye movement preparation. Indeed, the activation of the frontal, parietal, striatal and thalamic regions increases progressively during childhood [32]. Consequently, in order to further explore the relationship between latency and vergence deficiencies, given the considerable variability of this parameter, the examination of a population of children of similar age and with similar vertigo and vergence abnormalities, as reported in this study, is necessary.

The present study discloses a specific deficit: abnormally long mean latency values for eye movements for group 2, namely children with vertigo and an intermediate level of vergence abnormalities. This result is new and it expands our knowledge of oculomotor behavior in such children. Indeed, our previous study [3] of a small number of children (from 6 to 15 years old) with vergence abnormalities showed an overall abnormally long mean latency of eye movements, but the great variability in the ages of the children examined (6 to 15 years old) and the absence of the kind of vergence abnormality classification used here (see Table 1A, page 206) prevent us from validly comparing the present data with those obtained previously. We suggest that the latency abnormality reported for the majority of the children examined in our previous study [3] was most likely due to their different ages and to the different types of vergence abnormalities (several subjects had divergence abnormalities) of the children tested.

Finally, it should be noted that, compared to our previous work [3], the present report allows for better identification of vergence deficiencies and specific abnormalities in eye movement latencies; such characteristics could improve the diagnosis and screening of children suffering from vergence abnormalities.

Why were the latency values abnormal for children in group 2 only? As in our previous study of the consequences of vergence abnormalities for postural control in a similar population [7], children with intermediate vergence abnormalities suffered from and complained about their vergence abnormalities more than the other two groups, most likely because they had some remaining vergence abilities. For this group of children, correct vergence input is necessary to perform eye movements with normal latency, while the other two groups of children (with low and high vergence abnormalities) had already developed compensatory mechanisms to override impaired vergence information. Unfortunately, we do not have objective evidence on how such compensatory mechanisms have been developed but only subjective information. For instance, the group 1 could use its fusional vergence resources while the group 3, that had very weak or absent vergence abilities, most likely developed visual strategies to maintain acceptable vision during the day life. This hypothesis is corroborated by their less frequent headaches complaints reported by these two groups of children (respectively, 46% and 58%) versus 78% of the headaches observed in the group 2.

### Accuracy

This study shows that, in children with vertigo and vergence abnormalities, the accuracy of eye movements, in terms of both direction and depth, is abnormally poor. This finding confirms and extends our previous study [4] of a small group of children with vertigo, which showed that only vergence accuracy was abnormally poor. We suggest that the children examined here show a general difficulty localizing a target in space, whether the task calls for saccades or for vergence. This is in line with previous studies showing poor spatial discrimination in children with poor vergence capabilities [33].

### Peak velocity

In children with vertigo symptoms and vergence abnormalities, the peak velocity of eye movements in 3D space is similar to that of normal children. This finding suggests that the brain stem structures [8] controlling saccade and vergence dynamics in such children work as well as in normal children.

### Clinical considerations

This study revealed that vertigo symptoms and vergence abnormalities (statistically evaluated), which are becoming increasingly frequent in children, perhaps because of prolonged use of computer screens and video games [2],[34],[35], are associated with abnormal oculomotor performance, namely poor precision of eye movements in natural space. Importantly, this report also showed that a particular state of vergence abnormalities is responsible for abnormally long eye movements. Indeed, we were able to isolate a subgroup of children with vergence abnormalities assessed by static orthoptic tests, who showed signs of a specific impairment of structures controlling eye movements. Indeed, it is well known that in patients with cortical lesions latencies increase because of cortical dysfunction [36].

On the other hand, we have to point out that poor eye movements accuracy reported from the present study could be also caused by a reduced ability of our children to localize the target in depth correctly. This hypothesis is in agreement with previous clinical observations [33], [37]. Indeed, these authors observed that the performances in clinical tests assessing spatial discrimination (a synoptophore based vergence test, the Dunlop test) were significantly worse in children with poor vergence control than in normals. We believe that such impairment in the localization of the target could cause difficulty in the preparation and in the execution of vergence movements, leading to both abnormal longer latency and inaccurate eye movements.

Thus, our findings according to our previous studies [3], [4], [5] suggest the presence of a central/cortical impairment of the structures controlling the programming and the triggering of eye movements.

Finally, this type of research could have clinical implications for improving the screening of children who suffer from vergence abnormalities; it is also important for developing new training techniques to improve vergence eye movements with the implementation of visual attentional paradigms, such as the gap, which is generally used in basic research, given that such paradigms facilitate the programming of eye movements. Indeed, several studies showed the benefit of the orthoptic vergence training in subjects with vergence abnormalities [38], [39], [40]. Recently, also our group [3], [4], [5] reported after training an improvement of both objective eye movements performances as well as disappearance of subjective symptoms of vertigo and headaches. According to Fischer and Hartnegg [41], we suggest that training could help subjects to reinforce the visual perception of the target in the space and attentional mechanisms may also be responsible of such better perception and target localisation. Further research on this issue is needed.
